# Crystal structure of a mononuclear Ru^II^ complex with a back-to-back terpyridine ligand: [RuCl(bpy)(tpy–tpy)]^+^

**DOI:** 10.1107/S2056989015014632

**Published:** 2015-08-12

**Authors:** Francisca N. Rein, Weizhong Chen, Brian L. Scott, Reginaldo C. Rocha

**Affiliations:** a Los Alamos National Laboratory, Los Alamos, NM 87545, USA

**Keywords:** crystal structure, π–π stacking, terpyridine, ruthenium catalysts

## Abstract

We report the structural characterization of [6′,6′′-bis­(pyridin-2-yl)-2,2′:4′,4′′:2′′,2′′′-quaterpyridine](2,2′-bi­pyridine)­chlorido­ruthenium(II) hexa­fluorido­phosphate, [RuCl(C_10_H_8_N_2_)(C_30_H_20_N_6_)]PF_6_, which contains the bidentate ligand 2,2′-bi­pyridine (bpy) and the tridendate ligand 6′,6′′-bis­(pyridin-2-yl)-2,2′:4′,4′′:2′′,2′′′-quaterpyridine (tpy–tpy). The [RuCl(bpy)(tpy–tpy)]^+^ monocation has a distorted octa­hedral geometry at the central Ru^II^ ion due to the restricted bite angle [159.32 (16)°] of the tridendate ligand. The Ru-bound tpy and bpy moieties are nearly planar and essentially perpendicular to each other with a dihedral angle of 89.78 (11)° between the least-squares planes. The lengths of the two Ru—N bonds for bpy are 2.028 (4) and 2.075 (4) Å, with the shorter bond being opposite to Ru—Cl. For tpy–tpy, the mean Ru—N distance involving the outer N atoms *trans* to each other is 2.053 (8) Å, whereas the length of the much shorter bond involving the central N atom is 1.936 (4) Å. The Ru—Cl distance is 2.3982 (16) Å. The free uncoordinated moiety of tpy–tpy adopts a *trans,trans* conformation about the inter­annular C—C bonds, with adjacent pyridyl rings being only approximately coplanar. The crystal packing shows significant π–π stacking inter­actions based on tpy–tpy. The crystal structure reported here is the first for a tpy–tpy complex of ruthenium.

## Abstract 2

In this first crystal structure of an Ru complex with 6′,6"-bis­(pyridin-2-yl)-2,2′:4′,4":2",2"’-quaterpyridine, a ‘half’ of the ligand (one of the two terpyridyl units) is N^N^N *mer*-coordinated, whereas the other is free and adopts a *trans,trans* conformation about the inter­annular C—C bonds. The crystal packing features π–π stacking inter­actions between tpy–tpy ligands.

## Chemical context   

Aqueous homogeneous photocatalysis by supra­molecular assemblies is a powerful concept in the development of sunlight-driven catalytic schemes for renewable energy applications (Herrero *et al.*, 2011 ▸; Li *et al.*, 2012 ▸; Raynal *et al.*, 2014 ▸). In our recent efforts in this area, we have introduced alcohol-oxidation photocatalysts based on dinuclear Ru complexes (Chen *et al.*, 2009 ▸, 2011 ▸). One of these systems is the chromophore-catalyst dyad [(tpy)Ru(tpy–tpy)Ru(bpy)(H_2_O)]^4+^, in which the well-defined photosensitizer {(tpy)Ru(tpy)} and catalyst {(tpy)Ru(bpy)(H_2_O)} moieties are linked by the single covalent bond between the back-to-back terpyridines (tpy–tpy). In this and other related photocatalysts containing the {(tpy)Ru(bpy)(*L*)} moiety (*L* = H_2_O or Cl^−^), the aqua species is typically formed by easy ligand substitution from its chlorido precursor in water (Chen *et al.*, 2009 ▸; Davidson *et al.*, 2015 ▸; Jakubikova *et al.*, 2009 ▸; Li *et al.*, 2015 ▸). Therefore, the mononuclear chlorido complex **1** reported here was initially prepared and isolated as an inter­mediate in the synthesis of the dinuclear precatalyst [(tpy)Ru(tpy–tpy)Ru(bpy)(Cl)]^3+^ (Chen *et al.*, 2009 ▸). In addition to catalysis, the bridging tpy–tpy ligand finds relevance to the construction of donor–acceptor complexes with applications in charge/energy transfer and mol­ecular (opto)electronics (Wild *et al.*, 2011 ▸). Surprisingly, however, the crystal structure reported here is the first for an Ru^^II^^ complex.
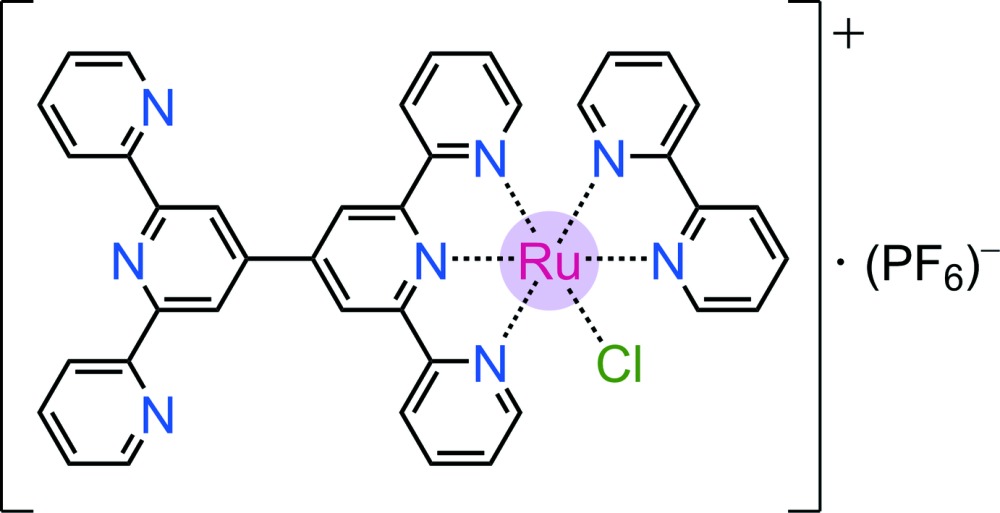


## Structural commentary   

**Figure 1:**
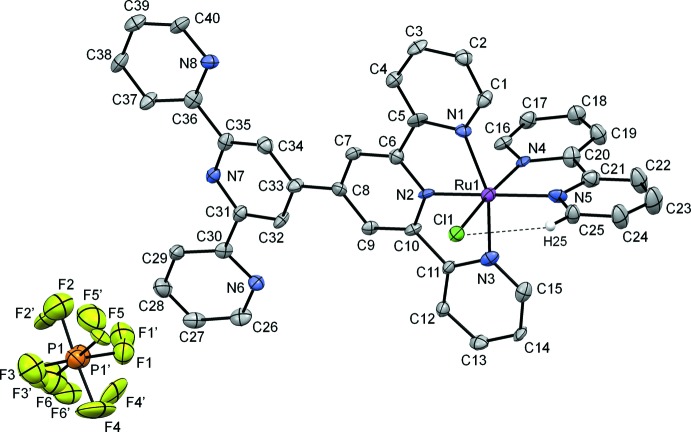
Single-crystal structure of **1**·PF_6_. Displacement ellipsoids are drawn at the 50% probability level. H atoms are omitted for clarity, except for H25.

The hexa­fluorido­phosphate salt of the monocationic complex (**1**·PF_6_) crystallizes in the triclinic (*P* 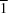) space group. The structure of **1** is shown in Figs. 1 ▸ and 2 ▸, and selected data are summarized in Table 1 ▸. The complex has a distorted octa­hedral geometry at the metal due to the restricted bite angle of its meridionally coordinating tridendate ligand (a tpy moiety). The N1—Ru—N3 angle of 159.32 (16)° is very similar to those of bis-terpyridyl Ru^II^ complexes (Chen *et al.*, 2013*a* ▸; Jude *et al.*, 2013 ▸), and far from the ideal angle of 180°. The bidentate bpy ligand has a *cis* configuration, with the N4—Ru—N5 angle of 79.04 (16)° in agreement with those found in similar chlorido Ru^II^-bpy complexes (Chen *et al.*, 2011 ▸, 2013*b* ▸). The N4 atom of bpy is arranged *trans* to the chlorido ligand in a nearly linear N4—Ru—Cl fashion [172.92 (12)°]. The Ru^II^ atom and atoms N2, N4, N5, and Cl1 form an equatorial plane with a maximum deviation of 0.032 (4) Å. The Ru-bound tpy moiety and bpy are approximately planar [with maximum deviations of 0.086 (5) Å and 0.071 (5) Å, respectively] and their mean planes are essentially perpendicular to each other with a dihedral angle of 89.78 (11)° between planes. For the tridentate ligand, the mean Ru—N distance involving the outer N1 and N3 atoms *trans* to each other is 2.053 (8) Å, whereas the bond distance involving the central N2 is much shorter [1.936 (4) Å] as a result of the structural constraint imposed by these *mer*-arranged ligands (Chen *et al.*, 2013*a* ▸; Jude *et al.*, 2013 ▸). For the bidentate ligand, the Ru—N distance is 2.075 (4) Å for N5 but only 2.028 (4) Å for N4, reflecting the increased Ru^II^→N_bpy_ π-backbonding inter­action at the coordinating atom *trans* to the π-donor Cl^−^ ligand (Chen *et al.*, 2013*b* ▸). The Ru—Cl distance of 2.3982 (16) Å is nearly the same as those observed previously (Chen *et al.*, 2013*b* ▸; Jude *et al.*, 2009 ▸). As expected, the free (uncoordinated) ‘half’ of tpy–tpy adopts a *trans,trans* conformation about the inter­annular C—C bonds (Constable *et al.*, 1993 ▸). Unlike the coordinating half of tpy–tpy, the rings of the free tpy moiety are only approximately coplanar, with angles of 20.9 (3)° and 13.3 (3)° between adjacent rings.

## Supra­molecular features   

**Figure 2:**
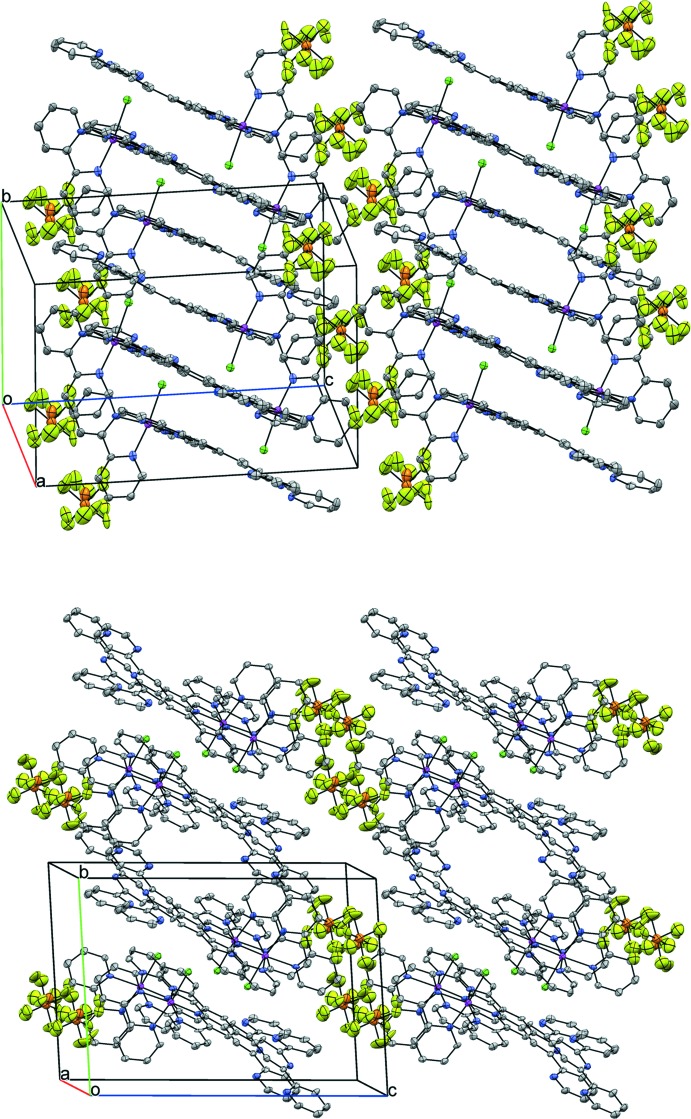
Two views of a 2×2×2 crystal packing diagram of **1**·PF_6_. Displacement ellipsoids are drawn at the 50% probability level. H atoms are omitted for clarity.

The intra­molecular Cl⋯H contact of 2.70 Å involving the hydrogen of the nearest C atom at bpy (H25) is similar to that observed earlier for complexes containing the {RuCl(bpy)} moiety (Chen *et al.*, 2011 ▸, 2013*b* ▸; Jude *et al.*, 2009 ▸). Although multiple inter­molecular and intra­molecular N⋯H distances that are shorter than the sum of van der Waals radii can be identified, the proximity appears to be mostly a consequence of geometry rather than chemically significant contacts. More relevant in the crystal packing of **1**·PF_6_ (Fig. 2 ▸) is the inter­molecular face-to-face π–π stacking between some of the pyridyl rings from tpy–tpy, for which the centroid–centroid distances (*Cg*⋯*Cg*) and plane–plane dihedral angles (α) are respectively: 3.723 (3) Å and 2.8 (2)° for (N3,C11,C12,C13,C14,C15)⋯(N1,C1,C2,C3,C4,C5) [sym­metry operation: −1 + *x*, *y*, *z*]; 3.812 (4) Å and 3.2 (2)° for (N3,C11,C12,C13,C14,C15)⋯(N2,C6,C7,C8,C9,C10) [sym­metry operation: 1–*x*, 1–*y*, 1–*z*]; 3.826 (4) Å and 5.6 (3)° for (N8,C36,C37,C38,C39,C40)⋯(N1,C1,C2,C3,C4,C5) [sym­metry operation: –*x*, –*y*, 1–*z*]; and 3.630 (4) Å and 15.5 (3)° for (N8,C36,C37,C38,C39,C40)⋯(N6,C26,C27,C28,C29,C30) [sym­metry operation: 1 + *x*, *y*, *z*]. In all these π–π stacking inter­actions, the slip angles from the parallel displacement (β, γ) are smaller than 30°.

## Database survey   

A search in the Cambridge Structural Database (Version 5.36; Groom & Allen, 2014 ▸) listed 50 hits for the tpy–tpy substructure; *i.e*. 6′,6′′-bis­(pyridin-2-yl)-2,2′:4′,4′′:2′′,2′′′-quaterpyridine. Other than one structure for the metal-free ligand itself (Constable *et al.*, 1993 ▸), one for an ytterbocene complex (Carlson *et al.*, 2006 ▸), and a few for Mn^II^ and Zn^II^ complexes (Koo *et al.*, 2003 ▸), all other structures are for Cu (mostly divalent) complexes and have been reported by Zubieta and colleagues (*e.g*. Koo *et al.*, 2003 ▸; Ouellette *et al.*, 2005 ▸; Jones *et al.*, 2013 ▸). The structure reported herein is thus the first for a tpy–tpy complex with a second-row transition metal ion.

## Synthesis and crystallization   

Compound **1**·PF_6_ was prepared by slow dropwise addition of a DMF solution of *cis*-Ru(bpy)(DMSO)_2_Cl_2_ into a solution of the tpy–tpy ligand (also in DMF) at reflux. The reaction solution was refluxed for another 2.5 h and then cooled down to room temperature. After evaporation of the solvent on a rotavap, water was added to dissolve the solid and excess NH_4_PF_6_ was added to form the precipitate, which was filtered off and dried under vacuum. Further purification was performed by column chromatography using alumina and a mixture of aceto­nitrile/toluene (1:2) as the eluant. The product was collected from the first band. The solvent was evaporated and the dark-red solid was collected and dried under vacuum (yield: 30%). Analysis calculated for C_40_H_28_N_8_F_6_PClRu: C, 53.25; H, 3.13; N, 12.42. Found: C, 52.71; H, 3.12; N, 11.86. Single crystals for X-ray structural analysis were grown by slow diffusion of diethyl ether into aceto­nitrile solutions of the complexes in long thin tubes.

## Other Characterization   

The identity of the complex [Ru(Cl)(bpy)(tpy–tpy)]^+^ was also characterized in MeCN solutions by other techniques. Mass spectra (ESI–MS: *m*/*z* 757) are in agreement with the formulation for the cation, *i.e*. [**1**(-PF_6_)]^+^ (calculated for C_40_H_28_N_8_ClRu, *m*/*z* 757.1). ^1^H-NMR (CD_3_CN, 400 MHz): *δ* 10.27–10.26 (*d*, 1H, aromatic), 9.07 (*s*, 2H, aromatic), 8.89 (*s*, 2H, aromatic), 8.73–6.95 (*m*, 23H, aromatic). Electrochemical measurements by cyclic voltammetry gave a redox potential of 0.83 V *vs* SCE for the reversible Ru^II^/Ru^III^ couple. This potential is anodically shifted by only 20 mV relative to the [Ru(Cl)(bpy)(tpy)]^+^ complex (0.81 V *vs* SCE; Chen *et al.*, 2009 ▸), which is consistent with the slightly more electron-withdrawing nature of tpy–tpy compared to tpy.

## Refinement   

Crystal data, data collection and structure refinement details are summarized in Table 2 ▸. All carbon-bound hydrogen-atom positions were idealized and set to ride on the atom they were attached to, with C—H = 0.93 Å (aromatic) and *U*_iso_(H) = 1.2*U*_eq_(C). Each atom in the anion was modeled in two positions, with site occupancies tied to 1.0. A total of 48 temperature-factor restraints were used to force convergence. The SQUEEZE routine in *PLATON* (van der Sluis & Spek, 1990 ▸; Spek, 2015 ▸) was used to treat disordered solvent mol­ecules. The given chemical formula and other crystal data do not take into account the solvent. The final refinement included anisotropic temperature factors on all non-hydrogen atoms.

## Supplementary Material

Crystal structure: contains datablock(s) I. DOI: 10.1107/S2056989015014632/pk2553sup1.cif

Structure factors: contains datablock(s) I. DOI: 10.1107/S2056989015014632/pk2553Isup2.hkl

CCDC reference: 1416756

Additional supporting information:  crystallographic information; 3D view; checkCIF report

## Figures and Tables

**Table 1:** Selected geometric parameters (, )

Ru1N2	1.936(4)	Ru1N5	2.075(4)
Ru1N4	2.028(4)	Ru1Cl1	2.3982(16)
Ru1N3	2.047(4)	C8C33	1.467(7)
Ru1N1	2.059(4)	Cl1H25	2.70
			
N2Ru1N4	96.32(17)	N3Ru1N5	97.77(16)
N2Ru1N3	79.86(16)	N1Ru1N5	102.89(16)
N4Ru1N3	92.26(16)	N2Ru1Cl1	90.73(12)
N2Ru1N1	79.48(16)	N4Ru1Cl1	172.92(12)
N4Ru1N1	90.79(15)	N3Ru1Cl1	89.60(12)
N3Ru1N1	159.32(16)	N1Ru1Cl1	89.87(12)
N2Ru1N5	174.75(17)	N5Ru1Cl1	93.94(12)
N4Ru1N5	79.04(16)		

**Table 2:** Experimental details

Crystal data	
Chemical formula	[RuCl(C_10_H_8_N_2_)(C_30_H_20_N_6_)]PF_6_
*M*_r_	902.19
Crystal system, space group	Triclinic, *P* 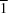
Temperature (K)	120
*a*, *b*, *c* ()	8.678(4), 13.743(7), 18.999(10)
, , ()	94.913(7), 90.583(7), 91.316(7)
*V* (^3^)	2257(2)
*Z*	2
Radiation type	Mo *K*
(mm^1^)	0.50
Crystal size (mm)	0.20 0.12 0.08

Data collection	
Diffractometer	Bruker D8 with APEXII CCD
Absorption correction	Multi-scan (*SADABS*; Bruker, 2007 ▸)
*T*_min_, *T*_max_	0.703, 0.961
No. of measured, independent and observed [*I* > 2(*I*)] reflections	22054, 8243, 4937
*R*_int_	0.109
(sin /)_max_ (^1^)	0.604

Refinement	
*R*[*F* ^2^ > 2(*F* ^2^)], *wR*(*F* ^2^), *S*	0.062, 0.136, 0.91
No. of reflections	8243
No. of parameters	578
No. of restraints	48
H-atom treatment	H-atom parameters constrained
_max_, _min_ (e ^3^)	0.74, 0.74

Computer programs: *APEX2* and *SAINT-Plus* (Bruker, 2007 ▸), *SHELXS97* and *SHELXL97* (Sheldrick, 2008 ▸), *Mercury* (Macrae *et al.*, 2008 ▸) and *publCIF* (Westrip, 2010 ▸).

## supplementary crystallographic information

### Crystal data

**Table:**

[RuCl(C_10_H_8_N_2_)(C_30_H_20_N_6_)]PF_6_	*Z* = 2
*M**_r_* = 902.19	*F*(000) = 908
Triclinic, *P*1	*D*_x_ = 1.328 Mg m^−^^3^
Hall symbol: -P 1	Mo *K*α radiation, λ = 0.71073 Å
*a* = 8.678 (4) Å	Cell parameters from 1124 reflections
*b* = 13.743 (7) Å	θ = 2.4–19.3°
*c* = 18.999 (10) Å	µ = 0.50 mm^−^^1^
α = 94.913 (7)°	*T* = 120 K
β = 90.583 (7)°	Block, red
γ = 91.316 (7)°	0.20 × 0.12 × 0.08 mm
*V* = 2257 (2) Å^3^	

### Data collection

**Table:**

Bruker D8 with APEXII CCD diffractometer	8243 independent reflections
Radiation source: fine-focus sealed tube	4937 reflections with *I* > 2σ(*I*)
Graphite monochromator	*R*_int_ = 0.109
ω scans	θ_max_ = 25.4°, θ_min_ = 1.9°
Absorption correction: multi-scan (*SADABS*; Bruker, 2007)	*h* = −10→10
*T*_min_ = 0.703, *T*_max_ = 0.961	*k* = −16→16
22054 measured reflections	*l* = −22→22

### Refinement

**Table:**

Refinement on *F*^2^	Primary atom site location: structure-invariant direct methods
Least-squares matrix: full	Secondary atom site location: difference Fourier map
*R*[*F*^2^ > 2σ(*F*^2^)] = 0.062	Hydrogen site location: inferred from neighbouring sites
*wR*(*F*^2^) = 0.136	H-atom parameters constrained
*S* = 0.91	*w* = 1/[σ^2^(*F*_o_^2^) + (0.0419*P*)^2^] where *P* = (*F*_o_^2^ + 2*F*_c_^2^)/3
8243 reflections	(Δ/σ)_max_ < 0.001
578 parameters	Δρ_max_ = 0.74 e Å^−^^3^
48 restraints	Δρ_min_ = −0.74 e Å^−^^3^

### Special details

**Table:**

Geometry. All e.s.d.'s (except the e.s.d. in the dihedral angle between two l.s. planes) are estimated using the full covariance matrix. The cell e.s.d.'s are taken into account individually in the estimation of e.s.d.'s in distances, angles and torsion angles; correlations between e.s.d.'s in cell parameters are only used when they are defined by crystal symmetry. An approximate (isotropic) treatment of cell e.s.d.'s is used for estimating e.s.d.'s involving l.s. planes.
Refinement. Refinement of *F*^2^ against ALL reflections. The weighted *R*-factor *wR* and goodness of fit *S* are based on *F*^2^, conventional *R*-factors *R* are based on *F*, with *F* set to zero for negative *F*^2^. The threshold expression of *F*^2^ > σ(*F*^2^) is used only for calculating *R*-factors(gt) *etc*. and is not relevant to the choice of reflections for refinement. *R*-factors based on *F*^2^ are statistically about twice as large as those based on *F*, and *R*-factors based on ALL data will be even larger.

### Fractional atomic coordinates and isotropic or equivalent isotropic displacement parameters (Å^2^)

**Table:**

	*x*	*y*	*z*	*U*_iso_*/*U*_eq_	Occ. (<1)
Ru1	0.31152 (5)	0.40649 (3)	0.31845 (2)	0.01950 (14)	
Cl1	0.20756 (14)	0.55446 (9)	0.37256 (6)	0.0221 (3)	
P1	0.0848 (11)	0.7198 (7)	0.9425 (5)	0.041 (2)	0.373 (15)
F1	0.185 (2)	0.6716 (13)	0.8753 (9)	0.052 (5)	0.373 (15)
F2	−0.011 (2)	0.6165 (12)	0.9447 (11)	0.083 (5)	0.373 (15)
F3	−0.009 (2)	0.7718 (12)	1.0054 (9)	0.071 (5)	0.373 (15)
F4	0.187 (3)	0.8171 (17)	0.9359 (14)	0.095 (9)	0.373 (15)
F5	−0.0441 (13)	0.7540 (13)	0.8891 (5)	0.049 (4)	0.373 (15)
F6	0.214 (2)	0.6793 (16)	0.9933 (11)	0.084 (7)	0.373 (15)
P1'	0.1274 (7)	0.6894 (5)	0.9376 (3)	0.0488 (16)	0.627 (15)
F1'	0.1952 (13)	0.6232 (9)	0.8730 (5)	0.075 (3)	0.627 (15)
F2'	0.0703 (14)	0.5948 (6)	0.9729 (4)	0.079 (4)	0.627 (15)
F3'	0.0586 (12)	0.7545 (7)	1.0043 (5)	0.068 (3)	0.627 (15)
F4'	0.1802 (16)	0.7844 (9)	0.9033 (7)	0.068 (4)	0.627 (15)
F5'	−0.0369 (10)	0.6894 (11)	0.8975 (5)	0.080 (3)	0.627 (15)
F6'	0.2860 (13)	0.6884 (6)	0.9794 (5)	0.066 (3)	0.627 (15)
N1	0.0981 (4)	0.3388 (3)	0.3224 (2)	0.0177 (10)	
N2	0.3256 (4)	0.3534 (3)	0.4094 (2)	0.0168 (10)	
N3	0.5281 (5)	0.4555 (3)	0.3497 (2)	0.0207 (10)	
N4	0.3947 (4)	0.2883 (3)	0.2611 (2)	0.0204 (10)	
N5	0.3130 (5)	0.4552 (3)	0.2180 (2)	0.0209 (10)	
N6	0.7959 (5)	0.2076 (3)	0.7126 (2)	0.0213 (10)	
N7	0.4133 (5)	0.1081 (3)	0.7186 (2)	0.0190 (10)	
N8	0.0356 (5)	0.0152 (3)	0.6655 (2)	0.0229 (10)	
C1	−0.0175 (5)	0.3340 (4)	0.2744 (3)	0.0223 (13)	
H1	−0.0029	0.3644	0.2330	0.027*	
C2	−0.1553 (6)	0.2869 (4)	0.2835 (3)	0.0230 (13)	
H2	−0.2316	0.2847	0.2486	0.028*	
C3	−0.1805 (6)	0.2418 (4)	0.3463 (3)	0.0241 (13)	
H3	−0.2733	0.2091	0.3535	0.029*	
C4	−0.0646 (5)	0.2471 (4)	0.3969 (3)	0.0218 (12)	
H4	−0.0786	0.2189	0.4393	0.026*	
C5	0.0717 (6)	0.2945 (3)	0.3839 (3)	0.0207 (12)	
C6	0.2055 (6)	0.3018 (4)	0.4340 (3)	0.0214 (12)	
C7	0.2167 (6)	0.2579 (3)	0.4963 (3)	0.0189 (12)	
H7	0.1332	0.2222	0.5118	0.023*	
C8	0.3525 (6)	0.2668 (4)	0.5362 (3)	0.0192 (12)	
C9	0.4728 (5)	0.3232 (3)	0.5111 (3)	0.0168 (11)	
H9	0.5643	0.3322	0.5369	0.020*	
C10	0.4565 (5)	0.3660 (3)	0.4476 (3)	0.0173 (12)	
C11	0.5715 (5)	0.4265 (4)	0.4147 (2)	0.0172 (12)	
C12	0.7154 (5)	0.4531 (4)	0.4451 (3)	0.0194 (12)	
H12	0.7434	0.4327	0.4888	0.023*	
C13	0.8163 (6)	0.5107 (4)	0.4085 (3)	0.0243 (13)	
H13	0.9124	0.5295	0.4279	0.029*	
C14	0.7733 (5)	0.5394 (4)	0.3439 (3)	0.0204 (12)	
H14	0.8403	0.5767	0.3186	0.024*	
C15	0.6282 (6)	0.5120 (4)	0.3169 (3)	0.0245 (13)	
H15	0.5986	0.5339	0.2739	0.029*	
C16	0.4309 (5)	0.2020 (4)	0.2859 (3)	0.0195 (12)	
H16	0.4197	0.1955	0.3339	0.023*	
C17	0.4820 (6)	0.1254 (4)	0.2448 (3)	0.0250 (13)	
H17	0.5046	0.0674	0.2642	0.030*	
C18	0.5005 (6)	0.1337 (4)	0.1735 (3)	0.0284 (14)	
H18	0.5340	0.0809	0.1442	0.034*	
C19	0.4695 (6)	0.2198 (4)	0.1462 (3)	0.0294 (14)	
H19	0.4849	0.2269	0.0986	0.035*	
C20	0.4152 (6)	0.2964 (4)	0.1900 (3)	0.0254 (13)	
C21	0.3745 (6)	0.3931 (4)	0.1671 (3)	0.0281 (14)	
C22	0.3947 (7)	0.4183 (4)	0.0986 (3)	0.0394 (16)	
H22	0.4368	0.3744	0.0645	0.047*	
C23	0.3512 (7)	0.5100 (4)	0.0819 (3)	0.0448 (17)	
H23	0.3677	0.5296	0.0369	0.054*	
C24	0.2835 (7)	0.5715 (4)	0.1324 (3)	0.0372 (16)	
H24	0.2482	0.6319	0.1216	0.045*	
C25	0.2687 (6)	0.5418 (4)	0.1998 (3)	0.0249 (13)	
H25	0.2254	0.5847	0.2343	0.030*	
C26	0.9244 (6)	0.2161 (4)	0.7534 (3)	0.0272 (14)	
H26	1.0148	0.2391	0.7342	0.033*	
C27	0.9280 (6)	0.1921 (4)	0.8225 (3)	0.0272 (13)	
H27	1.0188	0.1992	0.8490	0.033*	
C28	0.7979 (7)	0.1582 (4)	0.8509 (3)	0.0387 (16)	
H28	0.7984	0.1411	0.8972	0.046*	
C29	0.6637 (6)	0.1490 (4)	0.8110 (3)	0.0292 (14)	
H29	0.5727	0.1268	0.8303	0.035*	
C30	0.6661 (6)	0.1732 (3)	0.7422 (3)	0.0192 (12)	
C31	0.5239 (6)	0.1666 (4)	0.6984 (3)	0.0203 (12)	
C32	0.5088 (5)	0.2208 (4)	0.6396 (3)	0.0188 (12)	
H32	0.5906	0.2601	0.6266	0.023*	
C33	0.3710 (6)	0.2161 (4)	0.6005 (3)	0.0215 (12)	
C34	0.2524 (6)	0.1552 (4)	0.6246 (3)	0.0233 (13)	
H34	0.1567	0.1506	0.6019	0.028*	
C35	0.2801 (6)	0.1025 (4)	0.6822 (3)	0.0204 (12)	
C36	0.1559 (6)	0.0370 (4)	0.7088 (3)	0.0213 (12)	
C37	0.1701 (6)	0.0028 (4)	0.7753 (3)	0.0252 (13)	
H37	0.2561	0.0197	0.8037	0.030*	
C38	0.0555 (6)	−0.0559 (4)	0.7981 (3)	0.0306 (14)	
H38	0.0632	−0.0807	0.8420	0.037*	
C39	−0.0724 (6)	−0.0781 (4)	0.7552 (3)	0.0294 (14)	
H39	−0.1529	−0.1174	0.7696	0.035*	
C40	−0.0762 (6)	−0.0404 (4)	0.6909 (3)	0.0271 (13)	
H40	−0.1632	−0.0545	0.6625	0.032*	

### Atomic displacement parameters (Å^2^)

**Table:**

	*U*^11^	*U*^22^	*U*^33^	*U*^12^	*U*^13^	*U*^23^
Ru1	0.0195 (3)	0.0187 (3)	0.0207 (3)	−0.00196 (18)	0.00070 (18)	0.00520 (19)
Cl1	0.0220 (7)	0.0213 (8)	0.0231 (7)	−0.0007 (6)	0.0025 (6)	0.0030 (6)
P1	0.049 (3)	0.037 (3)	0.035 (3)	−0.002 (2)	−0.002 (2)	0.002 (2)
F1	0.059 (6)	0.057 (7)	0.041 (6)	0.000 (5)	0.008 (4)	0.008 (5)
F2	0.086 (7)	0.075 (7)	0.089 (7)	−0.019 (4)	0.010 (5)	0.009 (5)
F3	0.075 (7)	0.073 (7)	0.064 (6)	0.012 (5)	−0.002 (5)	0.001 (4)
F4	0.100 (12)	0.054 (12)	0.13 (2)	−0.049 (10)	−0.041 (15)	0.020 (12)
F5	0.053 (7)	0.066 (11)	0.025 (6)	−0.028 (7)	−0.011 (5)	−0.005 (6)
F6	0.094 (8)	0.088 (8)	0.072 (8)	0.008 (5)	−0.020 (5)	0.019 (5)
P1'	0.052 (2)	0.059 (3)	0.037 (2)	−0.0087 (19)	−0.0020 (18)	0.0111 (19)
F1'	0.100 (7)	0.075 (8)	0.050 (5)	−0.006 (6)	0.003 (4)	0.005 (6)
F2'	0.108 (8)	0.071 (6)	0.057 (5)	−0.063 (5)	0.000 (5)	0.020 (4)
F3'	0.071 (5)	0.085 (5)	0.044 (4)	−0.012 (4)	0.001 (4)	−0.012 (3)
F4'	0.060 (6)	0.070 (10)	0.082 (8)	−0.013 (6)	−0.005 (6)	0.064 (7)
F5'	0.067 (4)	0.103 (5)	0.068 (4)	−0.006 (4)	−0.012 (3)	−0.004 (4)
F6'	0.078 (7)	0.065 (5)	0.057 (5)	−0.027 (5)	−0.041 (5)	0.035 (4)
N1	0.014 (2)	0.019 (2)	0.021 (2)	−0.0010 (19)	−0.0042 (19)	0.002 (2)
N2	0.012 (2)	0.014 (2)	0.025 (2)	−0.0066 (18)	0.0028 (19)	0.0009 (19)
N3	0.021 (2)	0.011 (2)	0.031 (3)	0.0019 (19)	0.002 (2)	0.004 (2)
N4	0.014 (2)	0.028 (3)	0.019 (2)	−0.013 (2)	0.0024 (19)	0.003 (2)
N5	0.027 (3)	0.009 (2)	0.027 (3)	0.000 (2)	−0.003 (2)	0.000 (2)
N6	0.018 (2)	0.026 (3)	0.021 (2)	0.000 (2)	−0.0044 (19)	0.004 (2)
N7	0.019 (2)	0.018 (2)	0.020 (2)	0.0001 (19)	0.0030 (19)	0.0028 (19)
N8	0.021 (2)	0.020 (3)	0.027 (3)	−0.005 (2)	−0.005 (2)	0.003 (2)
C1	0.017 (3)	0.025 (3)	0.026 (3)	0.002 (2)	0.004 (2)	0.009 (3)
C2	0.022 (3)	0.022 (3)	0.025 (3)	0.001 (2)	−0.004 (2)	0.003 (3)
C3	0.010 (3)	0.025 (3)	0.038 (3)	−0.003 (2)	0.003 (2)	0.009 (3)
C4	0.019 (3)	0.022 (3)	0.025 (3)	−0.001 (2)	0.002 (2)	0.005 (2)
C5	0.023 (3)	0.005 (3)	0.036 (3)	0.002 (2)	−0.003 (3)	0.007 (2)
C6	0.024 (3)	0.019 (3)	0.021 (3)	−0.001 (2)	−0.001 (2)	0.003 (2)
C7	0.018 (3)	0.015 (3)	0.024 (3)	−0.004 (2)	0.001 (2)	0.004 (2)
C8	0.022 (3)	0.017 (3)	0.018 (3)	−0.003 (2)	0.001 (2)	−0.001 (2)
C9	0.018 (3)	0.010 (3)	0.023 (3)	0.001 (2)	−0.003 (2)	0.005 (2)
C10	0.012 (3)	0.015 (3)	0.025 (3)	−0.002 (2)	0.002 (2)	0.004 (2)
C11	0.017 (3)	0.018 (3)	0.018 (3)	−0.004 (2)	0.002 (2)	0.007 (2)
C12	0.021 (3)	0.021 (3)	0.017 (3)	−0.001 (2)	0.002 (2)	0.005 (2)
C13	0.014 (3)	0.027 (3)	0.031 (3)	−0.002 (2)	0.002 (2)	−0.003 (3)
C14	0.013 (3)	0.027 (3)	0.021 (3)	−0.006 (2)	0.006 (2)	0.007 (2)
C15	0.027 (3)	0.020 (3)	0.027 (3)	0.007 (3)	0.009 (3)	0.008 (3)
C16	0.014 (3)	0.017 (3)	0.028 (3)	−0.004 (2)	0.001 (2)	0.008 (3)
C17	0.030 (3)	0.020 (3)	0.027 (3)	−0.001 (3)	−0.002 (3)	0.010 (3)
C18	0.041 (4)	0.017 (3)	0.027 (3)	0.004 (3)	0.001 (3)	0.004 (3)
C19	0.042 (4)	0.024 (3)	0.022 (3)	0.007 (3)	0.000 (3)	0.000 (3)
C20	0.030 (3)	0.023 (3)	0.025 (3)	0.009 (3)	0.000 (3)	0.008 (3)
C21	0.033 (3)	0.021 (3)	0.030 (3)	−0.003 (3)	−0.005 (3)	0.005 (3)
C22	0.071 (5)	0.026 (4)	0.022 (3)	0.011 (3)	−0.001 (3)	0.005 (3)
C23	0.077 (5)	0.031 (4)	0.028 (4)	0.011 (4)	0.005 (3)	0.013 (3)
C24	0.062 (4)	0.027 (4)	0.025 (3)	0.009 (3)	−0.001 (3)	0.009 (3)
C25	0.037 (3)	0.018 (3)	0.020 (3)	0.004 (3)	0.006 (3)	0.002 (2)
C26	0.015 (3)	0.029 (3)	0.038 (4)	0.004 (3)	0.000 (3)	0.001 (3)
C27	0.026 (3)	0.017 (3)	0.039 (4)	0.002 (3)	−0.015 (3)	0.008 (3)
C28	0.051 (4)	0.039 (4)	0.027 (3)	−0.013 (3)	−0.013 (3)	0.012 (3)
C29	0.035 (3)	0.032 (4)	0.022 (3)	−0.014 (3)	−0.005 (3)	0.010 (3)
C30	0.023 (3)	0.011 (3)	0.025 (3)	0.003 (2)	−0.004 (2)	0.007 (2)
C31	0.021 (3)	0.017 (3)	0.023 (3)	0.001 (2)	0.004 (2)	0.004 (2)
C32	0.011 (3)	0.022 (3)	0.024 (3)	0.000 (2)	0.003 (2)	0.011 (2)
C33	0.016 (3)	0.022 (3)	0.027 (3)	−0.003 (2)	−0.001 (2)	0.004 (2)
C34	0.022 (3)	0.026 (3)	0.022 (3)	0.001 (2)	−0.002 (2)	0.000 (3)
C35	0.024 (3)	0.015 (3)	0.023 (3)	0.005 (2)	0.007 (2)	0.004 (2)
C36	0.024 (3)	0.015 (3)	0.026 (3)	0.008 (2)	0.000 (2)	0.006 (2)
C37	0.016 (3)	0.030 (3)	0.032 (3)	−0.003 (2)	−0.001 (2)	0.014 (3)
C38	0.030 (3)	0.030 (4)	0.034 (3)	−0.005 (3)	0.012 (3)	0.011 (3)
C39	0.019 (3)	0.028 (3)	0.043 (4)	−0.006 (3)	0.004 (3)	0.008 (3)
C40	0.023 (3)	0.021 (3)	0.037 (4)	−0.006 (3)	0.006 (3)	0.006 (3)

### Geometric parameters (Å, º)

**Table:**

Ru1—N2	1.936 (4)	C4—C5	1.372 (6)
Ru1—N4	2.028 (4)	C5—C6	1.490 (7)
Ru1—N3	2.047 (4)	C6—C7	1.378 (6)
Ru1—N1	2.059 (4)	C7—C8	1.391 (6)
Ru1—N5	2.075 (4)	C8—C9	1.399 (6)
Ru1—Cl1	2.3982 (16)	C8—C33	1.467 (7)
P1—F3	1.585 (17)	C9—C10	1.393 (6)
P1—F4	1.60 (2)	C10—C11	1.464 (6)
P1—F5	1.608 (14)	C11—C12	1.398 (6)
P1—F6	1.61 (2)	C12—C13	1.398 (6)
P1—F2	1.630 (17)	C13—C14	1.372 (7)
P1—F1	1.652 (19)	C14—C15	1.386 (7)
P1'—F4'	1.569 (12)	C16—C17	1.342 (7)
P1'—F6'	1.583 (10)	C17—C18	1.379 (7)
P1'—F2'	1.585 (8)	C18—C19	1.364 (7)
P1'—F1'	1.590 (11)	C19—C20	1.379 (7)
P1'—F5'	1.609 (10)	C20—C21	1.484 (7)
P1'—F3'	1.615 (10)	C21—C22	1.386 (7)
N1—C1	1.345 (6)	C22—C23	1.385 (7)
N1—C5	1.381 (6)	C23—C24	1.369 (8)
N2—C10	1.341 (6)	C24—C25	1.384 (7)
N2—C6	1.357 (6)	C26—C27	1.381 (7)
N3—C15	1.345 (6)	C27—C28	1.347 (7)
N3—C11	1.381 (6)	C28—C29	1.381 (7)
N4—C16	1.355 (6)	C29—C30	1.375 (7)
N4—C20	1.378 (6)	C30—C31	1.478 (7)
N5—C25	1.331 (6)	C31—C32	1.401 (7)
N5—C21	1.357 (6)	C32—C33	1.399 (6)
N6—C26	1.349 (6)	C33—C34	1.415 (6)
N6—C30	1.358 (6)	C34—C35	1.386 (7)
N7—C31	1.319 (6)	C35—C36	1.507 (7)
N7—C35	1.337 (6)	C36—C37	1.392 (7)
N8—C36	1.334 (6)	C37—C38	1.364 (7)
N8—C40	1.341 (6)	C38—C39	1.382 (7)
C1—C2	1.366 (6)	C39—C40	1.368 (7)
C2—C3	1.408 (7)	Cl1—H25	2.70
C3—C4	1.382 (7)		
			
N2—Ru1—N4	96.32 (17)	C1—C2—C3	119.2 (5)
N2—Ru1—N3	79.86 (16)	C4—C3—C2	118.6 (5)
N4—Ru1—N3	92.26 (16)	C5—C4—C3	119.0 (5)
N2—Ru1—N1	79.48 (16)	C4—C5—N1	122.9 (5)
N4—Ru1—N1	90.79 (15)	C4—C5—C6	123.3 (5)
N3—Ru1—N1	159.32 (16)	N1—C5—C6	113.8 (4)
N2—Ru1—N5	174.75 (17)	N2—C6—C7	121.4 (5)
N4—Ru1—N5	79.04 (16)	N2—C6—C5	112.1 (4)
N3—Ru1—N5	97.77 (16)	C7—C6—C5	126.4 (5)
N1—Ru1—N5	102.89 (16)	C6—C7—C8	120.3 (5)
N2—Ru1—Cl1	90.73 (12)	C7—C8—C9	117.2 (5)
N4—Ru1—Cl1	172.92 (12)	C7—C8—C33	121.5 (4)
N3—Ru1—Cl1	89.60 (12)	C9—C8—C33	121.2 (4)
N1—Ru1—Cl1	89.87 (12)	C10—C9—C8	120.5 (4)
N5—Ru1—Cl1	93.94 (12)	N2—C10—C9	120.7 (4)
F3—P1—F4	91.2 (13)	N2—C10—C11	112.6 (4)
F3—P1—F5	88.1 (8)	C9—C10—C11	126.8 (4)
F4—P1—F5	91.9 (11)	N3—C11—C12	121.5 (4)
F3—P1—F6	94.5 (10)	N3—C11—C10	114.7 (4)
F4—P1—F6	90.2 (13)	C12—C11—C10	123.7 (4)
F5—P1—F6	176.6 (10)	C11—C12—C13	118.7 (5)
F3—P1—F2	93.3 (9)	C14—C13—C12	119.8 (5)
F4—P1—F2	175.5 (13)	C13—C14—C15	118.9 (5)
F5—P1—F2	88.8 (8)	N3—C15—C14	123.4 (5)
F6—P1—F2	88.8 (10)	C17—C16—N4	123.5 (5)
F3—P1—F1	176.8 (10)	C16—C17—C18	119.2 (5)
F4—P1—F1	85.9 (11)	C19—C18—C17	119.6 (5)
F5—P1—F1	90.6 (9)	C18—C19—C20	119.4 (5)
F6—P1—F1	87.0 (10)	N4—C20—C19	121.3 (5)
F2—P1—F1	89.6 (9)	N4—C20—C21	113.8 (5)
F4'—P1'—F6'	90.7 (6)	C19—C20—C21	124.9 (5)
F4'—P1'—F2'	178.6 (7)	N5—C21—C22	122.1 (5)
F6'—P1'—F2'	90.2 (5)	N5—C21—C20	114.9 (5)
F4'—P1'—F1'	90.7 (6)	C22—C21—C20	123.0 (5)
F6'—P1'—F1'	91.1 (6)	C23—C22—C21	118.8 (5)
F2'—P1'—F1'	90.4 (5)	C24—C23—C22	119.3 (5)
F4'—P1'—F5'	90.8 (6)	C23—C24—C25	118.6 (5)
F6'—P1'—F5'	178.0 (6)	N5—C25—C24	123.5 (5)
F2'—P1'—F5'	88.4 (5)	N6—C26—C27	123.1 (5)
F1'—P1'—F5'	90.2 (6)	C28—C27—C26	119.0 (5)
F4'—P1'—F3'	90.5 (6)	C27—C28—C29	119.7 (5)
F6'—P1'—F3'	88.4 (6)	C30—C29—C28	119.2 (5)
F2'—P1'—F3'	88.3 (5)	N6—C30—C29	122.1 (5)
F1'—P1'—F3'	178.7 (6)	N6—C30—C31	117.2 (4)
F5'—P1'—F3'	90.3 (5)	C29—C30—C31	120.7 (5)
C1—N1—C5	116.9 (4)	N7—C31—C32	122.7 (5)
C1—N1—Ru1	128.9 (3)	N7—C31—C30	116.0 (4)
C5—N1—Ru1	114.2 (3)	C32—C31—C30	121.2 (4)
C10—N2—C6	119.9 (4)	C33—C32—C31	120.2 (5)
C10—N2—Ru1	119.7 (3)	C32—C33—C34	116.0 (5)
C6—N2—Ru1	120.5 (3)	C32—C33—C8	122.3 (5)
C15—N3—C11	117.7 (4)	C34—C33—C8	121.6 (5)
C15—N3—Ru1	129.3 (4)	C35—C34—C33	119.4 (5)
C11—N3—Ru1	113.0 (3)	N7—C35—C34	123.3 (5)
C16—N4—C20	116.9 (4)	N7—C35—C36	116.2 (4)
C16—N4—Ru1	126.3 (3)	C34—C35—C36	120.5 (5)
C20—N4—Ru1	116.8 (3)	N8—C36—C37	123.5 (5)
C25—N5—C21	117.6 (4)	N8—C36—C35	116.5 (4)
C25—N5—Ru1	127.0 (3)	C37—C36—C35	120.0 (5)
C21—N5—Ru1	115.3 (3)	C38—C37—C36	118.7 (5)
C26—N6—C30	116.9 (4)	C37—C38—C39	119.3 (5)
C31—N7—C35	118.3 (4)	C40—C39—C38	117.6 (5)
C36—N8—C40	115.7 (5)	N8—C40—C39	125.1 (5)
N1—C1—C2	123.3 (5)		
